# Targeted Epstein–Barr virus capture sequencing identifies BBLF4-L322M as an independent prognostic variant in nasopharyngeal carcinoma

**DOI:** 10.1128/spectrum.02927-25

**Published:** 2026-04-30

**Authors:** Shi Luo, Zongjian Huang, Nan Shi, Fangfang Chen, Weiren Xiang, Yu Ren, Wei Xia, Anzhou Tang

**Affiliations:** 1 Department of Otorhinolaryngology Head and Neck Surgery, The First Affiliated Hospital of Guangxi Medical University 117742, Nanning, Guangxi, China; 2 Key Laboratory of Early Prevention and Treatment for Regional High-Frequency Tumours (Guangxi Medical University), Ministry of Education 74626, Nanning, Guangxi, China; 3 Department of Otorhinolaryngology and Head and Neck Surgery, The Affiliated Zhuzhou Hospital Xiangya Medical College CSU, Zhuzhou, China; Penn State College of Medicine, Hershey, Pennsylvania, USA

**Keywords:** Epstein–Barr virus (EBV), nasopharyngeal carcinoma (NPC), viral prognostic biomarkers, capture-based whole-genome sequencing, BBLF4-L322M

## Abstract

Epstein–Barr virus (EBV) is etiologically linked to nasopharyngeal carcinoma (NPC) and shows geographic heterogeneity. To determine whether viral genotype affects prognosis, we performed capture-based whole-genome sequencing of EBV from 114 Guangxi NPC biopsies (mean breadth ~93%, depth ~979×) with Sanger confirmation at BNRF1-G696R. We detected 5,683 high-confidence variants with a mean of 1,112 per genome enriched in latent genes (LMP1, LMP2A/B, and the EBNA family). Variants with minor allele frequency ≥0.15 and missingness ≤0.05 were tested for association with 5-year overall survival. A total of 46 loci were significant in the univariate Cox models, and 19 missense variants underwent unsupervised clustering by genotype correlation, yielding two blocks. From these, BZLF1-A205S and BBLF4-L322M were selected as the index variants. In multivariable Cox models adjusted for age and dichotomized American Joint Committee on Cancer (AJCC) stage, BBLF4-L322M remained independently associated with poorer overall survival (OS), whereas BZLF1-A205S was not significant. A 720-genome phylogeny integrating our sequences with public data showed several Chinese sublineages with partial regional structuring. Guangxi strains were closely related to, yet partly distinct from, Guangdong and Hong Kong. These results support the incorporation of the EBV genotype, particularly BBLF4-L322M, into prognostic models and motivate functional studies to define mechanisms and validate their clinical utility.

**IMPORTANCE:**

The prognostic Epstein–Barr virus (EBV) variants in nasopharyngeal carcinoma (NPC) remain largely unknown. This study presents the first genome-wide survey of EBV sequence diversity in patients with NPC in Guangxi, China. The identified mutations, including BZLF1-A205S and BBLF4-L322M, are candidate viral biomarkers linked to patient survival. The abundance of nonsynonymous variants in latent phase genes implies a role for the viral genotype in the disease course. These insights have laid the groundwork for EBV-based prognostic biomarkers and precise medical strategies tailored to endemic NPC populations.

## INTRODUCTION

Nasopharyngeal carcinoma (NPC) has pronounced geographic and ethnic heterogeneity, with over 70% of the cases occurring in East and Southeast Asia (1). In endemic settings, Epstein–Barr virus (EBV) is consistently detected within the malignant epithelium, underscoring its etiological role (2). Recent genome-wide EBV studies have linked certain viral variants to the risk of NPC. Individuals carrying an EBV subtype defined by two linked BALF2 mutations (haplotype BALF2_CCT) have an approximately 11-fold higher risk of developing NPC than those carrying low-risk strains (3). Polymorphisms in the viral EBER noncoding RNA region are strongly associated with NPC, with one four-base deletion downstream of EBER2 occurring in ~97% of NPC biopsies; however, only in ~40% of healthy carriers (4). The circulating EBV DNA load in patient plasma correlates with tumor stage and prognosis (5). Collectively, these findings imply that EBV genetic features can significantly influence NPC progression and outcomes. However, studies directly linking specific EBV sequence variants to clinical prognosis, such as overall survival, remain limited. In this study, we aimed to identify EBV genomic markers predictive of NPC outcomes using whole-genome sequencing.

EBV also has a distinct geographic genetic structure. The virus is broadly classified into two types (I and II) based on the EBNA gene polymorphisms. Type I EBV is predominant in East Asia, including southern China and Europe. Meanwhile, type II EBV is more common in parts of Africa (6). High-resolution population genomics showed distinct EBV lineages and subpopulations clustered by region (7). Multiple high-incidence NPC regions harbor EBV sublineages that are enriched in patients with NPC and carry unique host-adapted markers. The phylogeographic structure of EBV suggests its co-evolution with human hosts and implies that region-specific viral variants may contribute to NPC risk.

The Guangxi Zhuang Autonomous Region of China exemplifies a high-NPC risk population, and its annual incidence is on the order of 20–40 cases per 100,000 people (8). Although some genomic studies have included NPC samples from Guangdong and Guangxi, the EBV population structure in Guangxi has not been systematically analyzed. The NPC epidemiology of Guangxi makes it an ideal setting for studying the genetic diversity and disease associations of EBV. To address this gap, we performed an EBV whole-genome phylogenetic analysis in Guangxi, focusing on viral variants linked to NPC prognosis and the local distribution of EBV subtypes. These data provide a genomic basis for understanding how EBV variability influences NPC clinical outcomes and for developing region-specific screening and treatment strategies.

## RESULTS

### Probe capture sequencing and annotation

**Fig 1:**
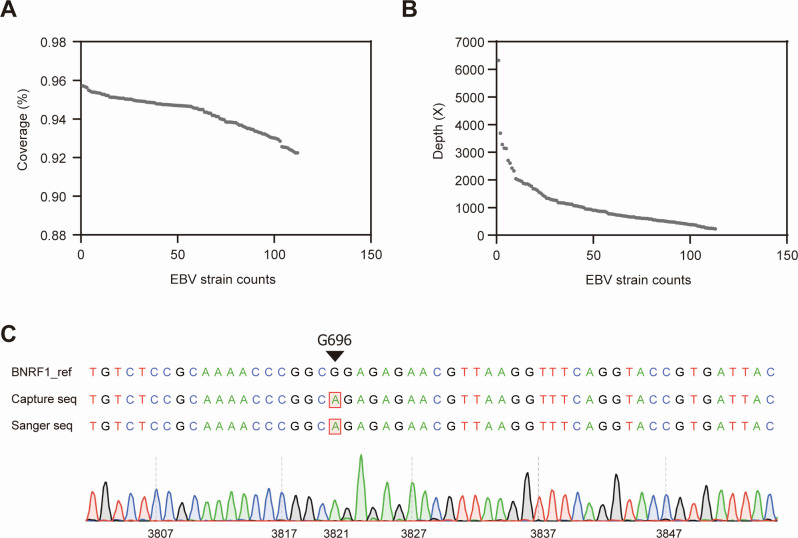
Probe capture sequencing and validation of EBV genomes from NPC tissues. (**A**) Genome coverage across EBV strains recovered from tumor specimens (*n* = 114). Each dot represents one strain ordered by decreasing coverage: mean coverage ≈ 93%. (**B**) Sequencing depth for the same strains ordered by decreasing depth; mean depth 979× (Table S1). (**C**) Validation of probe capture sequencing accuracy by comparison with Sanger sequencing at the BNRF1 G696R variant. Top, alignment of the EBV BNRF1 reference sequence with probe-capture and Sanger sequences (boxed bases mark the variant site). Bottom, representative Sanger chromatogram around the locus.

In this approach, biotinylated probes targeting the EBV genome were used to enrich viral DNA from the NPC biopsy samples before sequencing, enabling high coverage of the EBV genome. The clinical characteristics of the enrolled patients are summarized in Table 1. A total of 114 Epstein–Barr virus (EBV) strains were obtained, with an average coverage of approximately 93% (Fig. 1A), and an average depth of sequencing of 979× (Fig. 1B; Table S1). The accuracy was confirmed by Sanger sequencing of BNRF1-G696R, a variant previously associated with NPC risk (9). This showed complete concordance with the probe capture-derived sequences (Fig. 1C).

**TABLE 1:** Patient characteristics^*a*^

Characteristics	Group	Frequency	Ratio
Gender	Male	87	76.32%
	Female	27	23.68%
Age	Age < 50	65	57.02%
	Age ≥ 50	49	42.98%
EBV-EBNA1 IgA	++	51	44.74%
	＋	18	15.79%
	−	10	8.77%
	NA	35	30.70%
EBV-VCA IgA	++	19	16.67%
	＋	19	16.67%
	−	41	35.96%
	NA	35	30.70%
AJCC 8th Edition	I	1	0.88%
	II	11	9.65%
	III	29	25.44%
	IV	48	42.11%
	NA	25	21.93%
CLNM	Yes	84	73.68%
	No	7	6.14%
	NA	23	20.18%
Distant metastasis	Yes	10	8.77%
	No	68	59.65%
	NA	36	31.58%

^*a*^ EBV-EBNA1 IgA and EBV-VCA IgA titers were reported as semi-quantitative categories: +++, strongly positive; +, positive; − negative; NA, not available/not assessed. The 8th edition of the AJCC staging system denotes the stage at diagnosis. CLNM, cervical lymph node metastasis; distant metastasis recorded at diagnosis.

**Fig 2:**
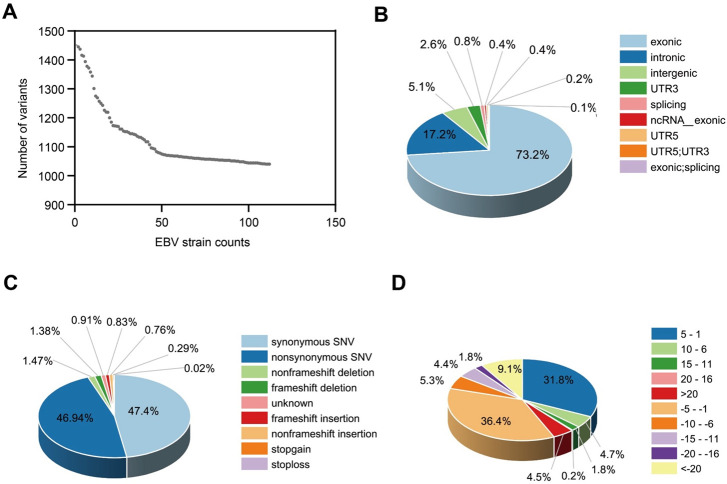
EBV variant landscape in NPC by probe-capture sequencing. (**A**) Variant burden per EBV strain: 5,683 SNPs/InDels after filtering (*n* = 114; mean 1,112 per sample). (**B**) Genomic distribution: 73.2% exonic, 17.2% intronic; remaining in intergenic/UTRs, with ≤1% at splice sites. (**C**) Functional classes: nonsynonymous 47.4%, synonymous 46.9%; frameshift and stop-gain/stop-loss events are rare. (**D**) Indel size: short events predominate, 1–5 bp deletions 36.4% and 1–5 bp insertions 31.8% (positive = insertions; negative = deletions).

After filtering, 5,683 SNP/InDel variants were detected, with a mean of 1,112 variants per sample (Fig. 2A). We examined the genomic distribution and functional categories of these variants. Most variants (73.2%) were located in exonic regions, where they could alter protein-coding sequences. An additional 17.2% occurred at exon–intron boundaries, implying a potential impact on RNA splicing, and consequently, protein synthesis (Fig. 2B). Although variants in the untranslated regions (UTRs) are less frequent, they may be involved in post-transcriptional regulation. Regarding the variant type (Fig. 2C), nonsynonymous substitutions formed a slightly larger proportion (47.4%), indicating the potential of amino acid alterations to influence protein function. Synonymous substitutions accounted for 46.9%. Despite not altering the encoded amino acids, these variants may affect gene expression through codon usage, mRNA secondary structure, or splicing regulation (10). Frameshift insertions and deletions were rare. Analysis of the indel size distribution showed that 36.4% were 1–5 bp deletions and 31.8% were 1–5 bp insertions. This indicated that short indels constitute most indel events in the EBV genome (Fig. 2D). These findings underscore the enrichment of exonic and splice-site mutations, the functional importance of nonsynonymous changes, and the adaptive role of short indels in EBV evolution and pathogenesis.

### EBV gene polymorphism

**Fig 3:**
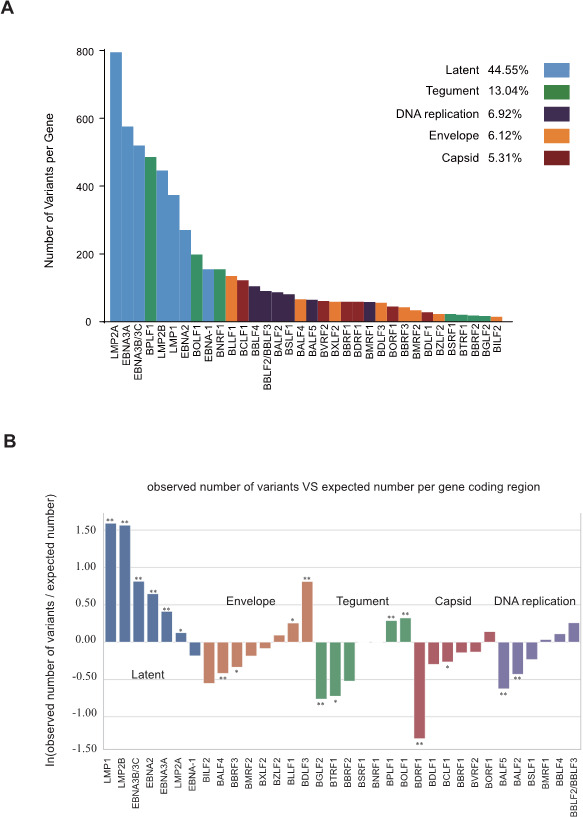
Gene-level distribution and enrichment of EBV polymorphisms. (**A**) Genes with ≥10 independent variants (*n* = 34; Table S2) were grouped into five functional classes. Bars show per-gene variant counts; category totals are indicated at right (Latent 44.6%, Tegument 13.0%, DNA replication 6.9%, Envelope 6.1%, and Capsid 5.3%). (**B**) Length-normalized enrichment (ln[observed/expected] per coding region). Latent genes exhibit the strongest excess of polymorphism, with LMP-1, LMP-2B, EBNA-3B/3C, EBNA-2, and EBNA-3A enriched (*P* < 0.01) and LMP-2A enriched (*P* < 0.05). Asterisks denote significance (*P < 0.05, **P < 0.01).

To characterize the polymorphic landscape of the EBV genome, we retained genes harboring at least 10 independent variants to minimize the sampling errors associated with sparsely mutated loci. Each gene was assigned to one of the five canonical functional categories, namely, latent, tegument, DNA-replication, envelope, or capsid. These criteria yielded 34 representative genes (Table S2). Seven of the 10 most variable genes (LMP-2A, LMP-2B, LMP-1, EBNA-3A, EBNA-3B/3C, EBNA-2, and EBNA-1) were latency-associated and accounted for 44.6% of all detected variants, significantly exceeding any other category (Fig. 3A). Length-normalized log-ratio analysis further showed that LMP-1, LMP-2B, EBNA-3B/3C, EBNA-2, and EBNA-3A harbored more polymorphisms than expected (*P*  < 0.01), and LMP-2A was enriched (*P*  < 0.05) (Fig. 3B). Latent genes constitute the densest polymorphic region of the EBV genome, likely reflecting immune-driven diversification during prolonged host persistence.

### dN/dS of EBV latent genes

We then quantified selective pressure on the six latent genes that contained coding-region substitutions (LMP-2A, LMP-1, EBNA-3A, EBNA-3B/3C, EBNA-2, and EBNA-1). LMP-2B was excluded because none of its variants fell within the open reading frame. For each gene, we computed the nonsynonymous (dN) and synonymous (dS) substitution rates and the resulting dN/dS ratios (Table 2). EBNA-3A, EBNA-3B/3C, LMP-1, EBNA-2, and EBNA-1 showed dN/dS > 1 (*P* < 0.05), indicating strong positive selection. This is consistent with the immune-mediated pressure that favors amino acid changes in the exposed or functional domains to facilitate viral persistence and transmission.

**TABLE 2:** dN/dS of EBV latent genes^*a*^

Gene	dN	dS	dN/dS
LMP-2A	41	44	0.93
EBNA-3A	200	99	2.02
EBNA-3B/EBNA-3C	300	165	1.82
LMP-1	178	67	2.66
EBNA-2	61	32	1.91
EBNA-1	48	14	3.43

^*a*^ EBNA, Epstein–Barr nuclear antigen; dN/dS, ratio of nonsynonymous (dN) to synonymous (dS) substitution rates, indicating selective pressure (dN/dS > 1 suggests positive selection, dN/dS < 1 suggests purifying selection); LMP, latent membrane protein.

### Identification of risk SNPs associated with NPC prognosis

To investigate the impacts of EBV genomic variations on NPC prognosis, univariate and multivariate Cox regression analyses were performed. We included EBV genomes from 114 patients with NPC with a minimum 5-year follow-up period. After quality control (minor-allele frequency ≥0.15; missing rate ≤0.05), 796 SNPs remained. Using univariate Cox regression analysis, 46 SNPs were found to be associated with overall survival. The nonsynonymous SNPs are listed in Table 3 (Table S3). Six high-frequency missense variants in BZLF1 (A205S, Q163L, V152A, T124P, S76P, and T68A; each present in ~80% of strains) were consistently protective with HRs ≈0.30–0.36 (e.g., BZLF1-A205S, HR = 0.297, 95% CI = 0.113–0.782, *P* = 0.014). Additional protective signals were observed for BRLF1-A377E (HR = 0.361, 95% CI = 0.137–0.948, *P* = 0.039), BRRF2-T374A (HR = 0.338, 95% CI = 0.129–0.888, *P* = 0.028), and BARF0 substitutions Q214E, R257C, and V264I (HRs ≈ 0.30-0.34, all *P* < 0.03). Conversely, several variants were associated with increased hazard, including BBLF4-L322M (HR = 3.871, 95% CI = 1.492–10.047, *P* = 0.005), BRRF2-A521T (HR = 3.405, 95% CI = 1.295–8.954, *P* = 0.013), BRRF2-S397P and BRRF2-L390S (both HR = 2.924, 95% CI = 1.112–7.686, *P* = 0.030), and BKRF4-P146L (HR = 3.182, 95% CI = 1.210-8.367, *P* = 0.019).

**TABLE 3:** Nonsynonymous EBV variants associated with overall survival (univariate Cox analysis)^*a*^

EBV variant	Gene	Allele frequency/*N* (%)	Gene region	HR (95% CI)	*P* value
c.613G>T/p.A205S	BZLF1	93/114 (81.58)	Exonic	0.297 (0.113–0.782)	0.014
c.488A>T/p.Q163L	BZLF1	91/114 (79.82)	Exonic	0.339 (0.129–0.891)	0.028
c.455T>C/p.V152A	BZLF1	92/114 (80.70)	Exonic	0.318 (0.121–0.836)	0.020
c.370A>C/p.T124P	BZLF1	91/114 (79.82)	Exonic	0.339 (0.129–0.891)	0.028
c.226T>C/p.S76P	BZLF1	92/114 (80.70)	Exonic	0.318 (0.121–0.836)	0.020
c.202A>G/p.T68A	BZLF1	92/114 (80.70)	Exonic	0.318 (0.121–0.836)	0.020
c.1130C>A/p.A377E	BRLF1	90/114 (78.95)	Exonic	0.361 (0.137–0.948)	0.039
c.1120A>G/p.T374A	BRRF2	91/114 (79.82)	Exonic	0.338 (0.129–0.888)	0.028
c.1154T>C/p.M385T	BKRF2	90/113 (79.65)	Exonic	0.342 (0.130–0.899)	0.030
c.1165C>A/p.H389N	BRRF2	23/113 (20.35)	Exonic	2.924 (1.112–7.686)	0.030
c.1169T>C/p.L390S	BRRF2	23/113 (20.35)	Exonic	2.924 (1.112–7.686)	0.030
c.1189T>C/p.S397P	BRRF2	23/113 (20.35)	Exonic	2.924 (1.112–7.686)	0.030
c.1561G>A/p.A521T	BRRF2	21/114 (18.42)	Exonic	3.405 (1.295–8.954)	0.013
c.437C>T/p.P146L	BKRF4	22/114 (19.30)	Exonic	3.182 (1.210–8.367)	0.019
c.964C>A/p.L322M	BBLF4	23/114 (20.18)	Exonic	3.871 (1.492–10.047)	0.005
c.640C>G/p.Q214E	BARF0	95/114 (83.33)	Exonic	0.328 (0.121–0.888)	0.028
c.649C>T/p.P217S	BARF0	18/114 (15.79)	Exonic	3.298 (1.218–8.929)	0.019
c.769C>T/p.R257C	BARF0	96/114 (84.21)	Exonic	0.303 (0.112–0.821)	0.019
c.790G>A/p.V264I	BARF0	96/114 (84.21)	Exonic	0.303 (0.112–0.821)	0.019

^*a*^ CI, confidence interval; EBV, Epstein–Barr virus; HR, hazard ratio. HR: the factor by which the hazard (risk of death) is multiplied for patients with the variant, compared to those without the variant.

**Fig 4:**
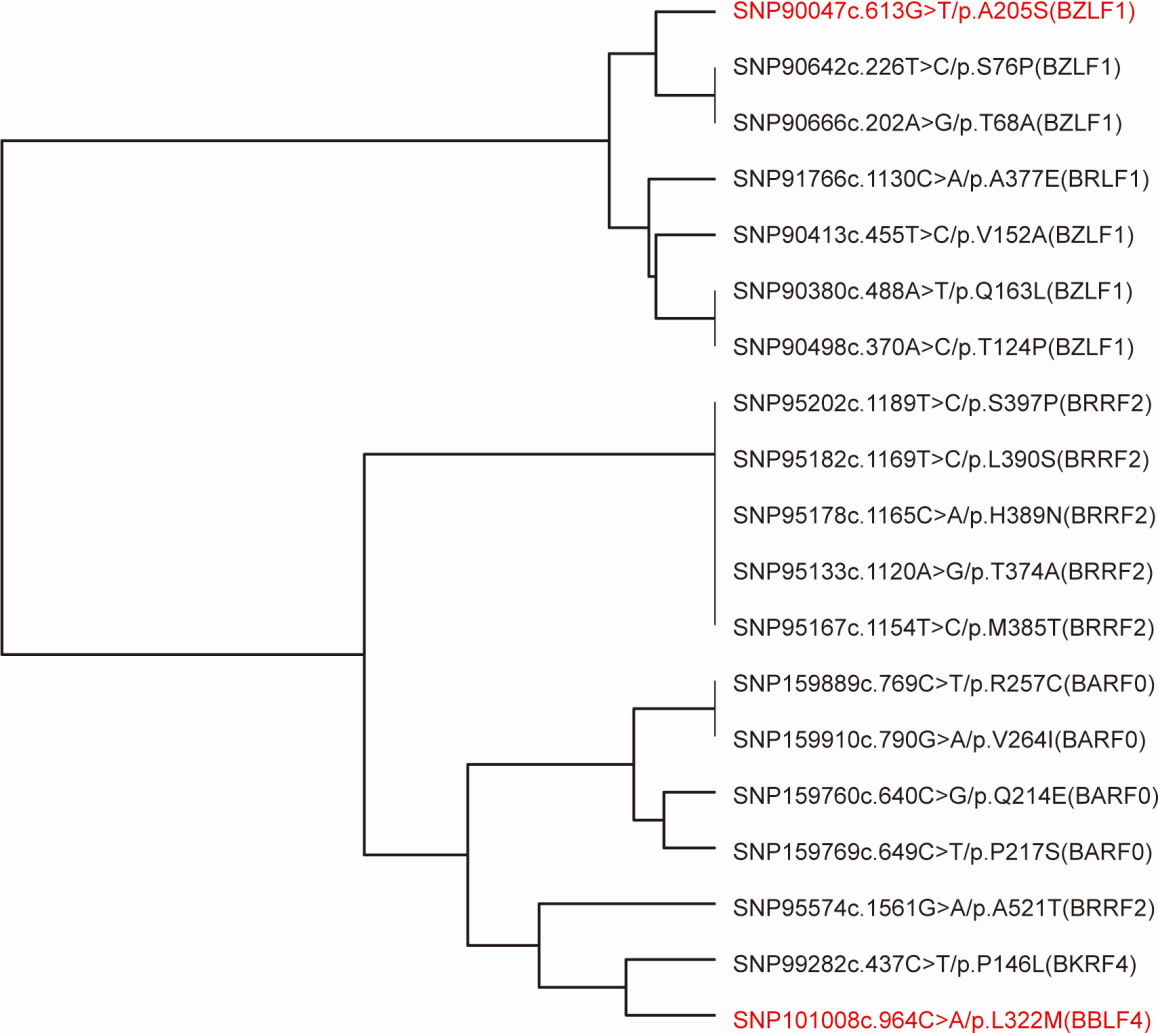
Unsupervised clustering of nonsynonymous EBV variants. Hierarchical agglomerative clustering of 114 EBV strains showed gene-centric variant modules. BZLF1 substitutions clustered together, as did the BRRF2 and BARF0 variants, indicating a coordinated occurrence within the genes. Two variants with distinct patterns (BZLF1 p.A205S and BBLF4 p.L322M) are highlighted in red for follow-up analysis.

To identify independent rather than redundant signals, we clustered 19 nonsynonymous candidates across the 114 EBV strains using pairwise genotype correlations. The resulting dendrogram delineated two distinct blocks (Fig. 4). From each block, we retained the variant with the smallest univariate *P* value as the representative marker SNP (SNP90047 (BZLF1-A205S) for block 1 and SNP101008 (BBLF4-L322M) for block 2) for the multivariable Cox model to minimize collinearity and preserve the strongest independent associations with survival. The multivariable Cox model was adjusted for clinical covariates. Given the skewed stage distribution (I: 1, II: 11, III: 29, and IV: 48), the AJCC stage was dichotomized as early (I–III) or late (IV). In the adjusted analysis, late stage remained a strong predictor of poor OS (HR = 10.93, 95% CI = 1.394–85.706; *P* = 0.0228). BBLF4-L322M was independently associated with higher mortality (HR = 2.931, 95% CI = 1.098–7.825; *P* = 0.0318). Meanwhile, the protective effect of BZLF1-A205S was attenuated and not significant (HR = 0.497, 95% CI = 0.181–1.365; *P* = 0.175)(Table 4). These results prioritize BBLF4-L322M as an EBV-independent risk variant for early mortality in NPC. Meanwhile, BZLF1-A205S appears to be protective in univariate analyses; however, it requires validation for independence in larger cohorts.

**TABLE 4:** Multivariable Cox model of EBV variants and clinical factors associated with survival^*a*^

EBV variant (gene)	HR (95% CI)	*P* value
SNP90047 c.613G>T/p.A205S(BZLF1)	0.497 (0.181–1.365)	0.175
SNP101008 c.964C>A/p.L322M(BBLF4)	2.931 (1.098–7.825)	0.032
Age	1.043 (0.993–1.096)	0.091
AJCClate^*a*^	10.93 (1.394–85.706)	0.023

^*a*^ AJCClate indicates AJCC 8th edition Stage IV versus Stages I–III; the stage was dichotomized owing to the skewed distribution. CI, confidence interval; HR, hazard ratio; NPC, nasopharyngeal carcinoma; OS, overall survival. HR: the factor by which the hazard (risk of death) is multiplied for patients with the variant, compared to those without the variant.

### Phylogenetic structure of EBV genomes across geography and phenotype

**Fig 5:**
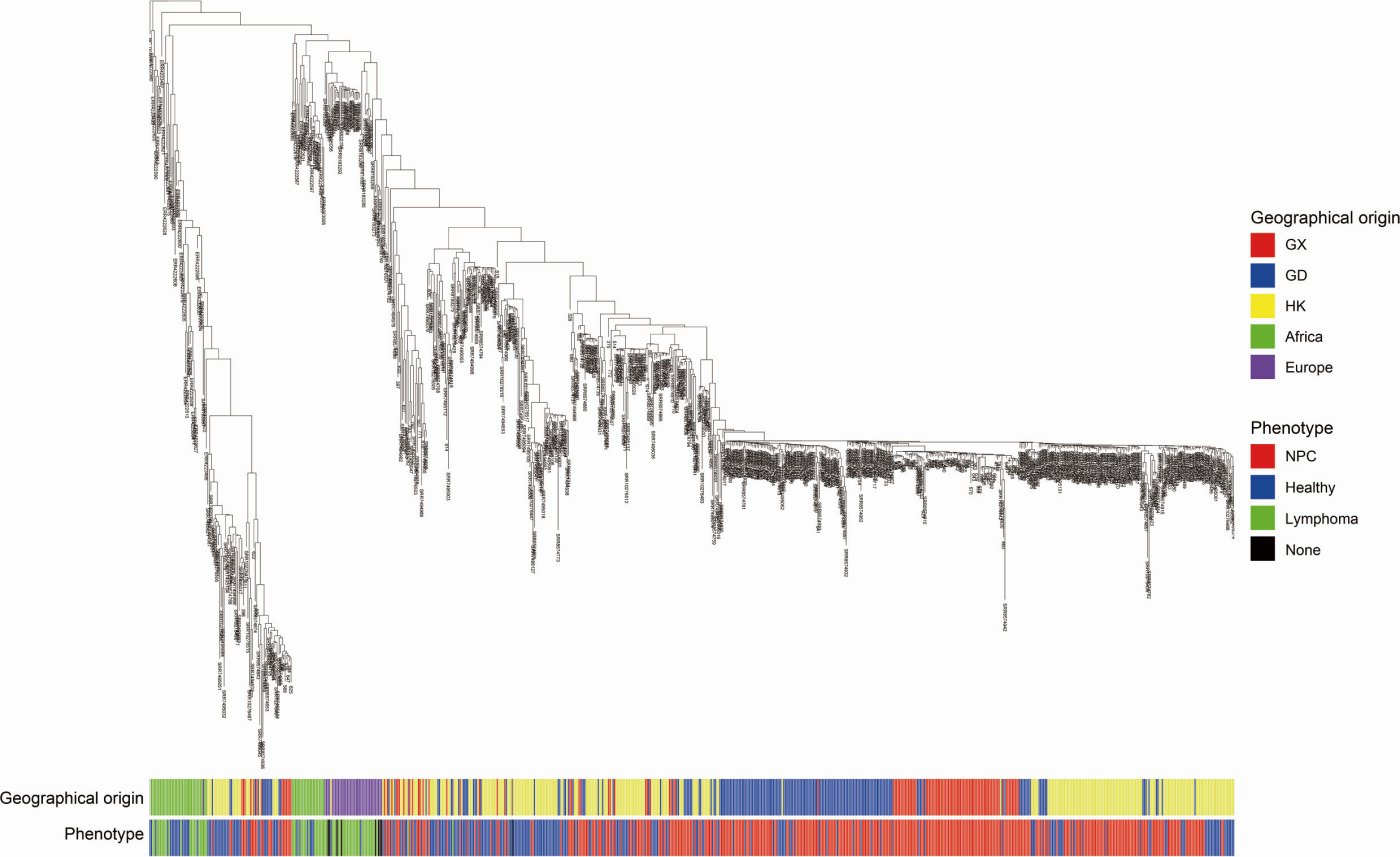
Whole-genome phylogeny of EBV with geographic and clinical phenotypes. Phylogeny inferred from genome-wide variant profiles of EBV isolates. The branch lengths reflect nucleotide divergence. Colored metadata tracks beneath the tree denote the geographical origins of each isolate: GX (Guangxi), GD (Guangdong), HK (Hong Kong), Africa, Europe, and the phenotypes (NPC, Healthy, Lymphoma, and None), as indicated in the legend.

We reconstructed a whole-genome phylogeny from the combined EBV data set (731 strains) and annotated each strain according to its geographical origin and disease phenotype (Fig. 5). The tree resolved multiple viral lineages, rather than a single dominant clade. Chinese strains, comprising those of Guangxi, Guangdong, and Hong Kong, accounted for most of the sequences and formed several large sublineages that showed partial geographic assortativity. Clusters were enriched for Hong Kong or Guangdong sequences, whereas African and European isolates tended to group toward discrete branches at the tree periphery. A similar pattern was evident for the disease phenotype: NPC genomes were more frequent in several Chinese sublineages, including GX-containing clades, indicating a tendency toward NPC enrichment. However, EBV sequences from healthy carriers and from lymphoma patients were interspersed throughout the tree. Within the NPC-derived genomes, the EBV strains from Hong Kong, Guangxi, and Guangdong occupied partially distinct sublineages, indicating region-specific prevalence patterns. The phylogeny suggests that geographic origin and clinical phenotype have a strong influence on the EBV population structure. To further assess the population distribution of BBLF4-L322M, we summarized its frequency across the comparative data set by region. The mutation was detected in Hong Kong (45/302, 14.90%), Guangxi (30/171, 17.54%), and Guangdong (17/146, 11.64%), but not in African (0/59) or European (0/42) genomes. These data indicate marked geographic structure and suggest that BBLF4-L322M is enriched in East Asian EBV lineages rather than representing a universal global variant. When grouped by disease category, the frequency of BBLF4-L322M was 10.18% in NPC (40/393), 20.88% in healthy carriers (52/249), and 0% in lymphoma/BL (0/69). Therefore, in the currently available public data set, BBLF4-L322M was not enriched in NPC relative to healthy carriers. This argues against interpreting BBLF4-L322M as an NPC-specific pathogenic mutation.

## DISCUSSION

In this study, whole-genome sequencing of EBV from Guangxi patients with NPC showed lineage-specific patterns of viral variation with potential clinical relevance. Genetic variants were highly enriched in latent-phase genes, such as LMP1, LMP2A/B, and EBNA family genes, which exhibited the highest SNP densities in the genome. Our dN/dS analysis further showed that multiple latent antigen genes were under positive selection, consistent with immune-driven EBV diversification. For latent proteins, such variation could influence immune regulation through several mechanisms (11). Because EBNA1 can limit antigen presentation and NK-cell recognition, sequence variation in EBNA1 could modify these effects (12, 13), whereas variation in LMP1 or LMP2A may alter checkpoint- and cytokine-related signaling and MHC expression (14, 15). More broadly, sequence variation in latent proteins could modify susceptibility of infected cells to T- and NK-cell cytotoxicity while also reshaping latency-associated oncogenic signaling (16). We therefore interpret the enrichment of latent-gene variation not only as a footprint of host immune selection but also as a plausible contributor to immune escape and tumor biology in NPC.

Short frameshift indels (1–5 bp) accounted for most of the observed mutations. This suggests that the EBV genome may have gained an evolutionary advantage through the accumulation of micro-indels. A large-scale EBV sequencing effort identified 8,015 SNPs and 454 indels across the viral genome, with latency genes being among the most polymorphic (3). Our finding of abundant small indels echoes this finding and suggests that indel variation is a common feature of EBV evolution in NPC.

A major innovation of our study is the association between EBV gene variations and NPC patient prognosis. In the Guangxi NPC cohort with follow-up, we identified several EBV variants that were significantly correlated with overall survival. A leucine-to-methionine substitution at position 322 in the EBV helicase gene BBLF4 (“L322M”) was a strong predictor of survival. This polymorphism was significantly enriched in both longer-surviving and shorter-surviving patients, including after adjusting for clinical stage. To date, most large-scale EBV genomic studies have focused on susceptibility loci rather than prognosis. Xu et al. identified two nonsynonymous BALF2 variants that together accounted for most of the NPC risk in southern China (3). Wong et al. (17) identified the same EBER2 deletion as the most common NPC-associated EBV variant in a recent meta-analysis (17). Zhang et al. (18) reported that high-risk EBV haplotypes are highly enriched in familial NPC (18). In our NPC cohort, BBLF4-L322M stood out after controlling for known factors. By contrast, BBLF4 is a lytic-cycle gene encoding the helicase component of the EBV helicase–primase complex. The prognostic association of BBLF4-L322M may therefore reflect altered viral replication biology rather than the same mechanisms inferred for latent genes. If L322M affects helicase–primase activity or complex assembly, it could change lytic DNA replication efficiency, progeny virion production, or the latent–lytic balance (19). Such changes could, in turn, alter antigen release, paracrine inflammatory signaling, and the local tumor microenvironment. At present, these possibilities remain mechanistic hypotheses and require direct functional testing.

Phylogenetic analysis of the sequences showed a clear geographical structure. Chinese NPC-derived EBV genomes did not form a single clade; however, several sublineages, with strains from Guangxi showing partial separation from those from Guangdong and Hong Kong. This mirrors recent population studies: Xue et al. found a “Chinese-unique” EBV cluster distinct from other global lineages (9), and Tu et al. reported that NPC biopsy-derived EBV from Hunan Province formed clades distinct from published reference strains (20). EBV genomes are highly diverse and are not strictly determined by the tumor type (21). A recent evolutionary analysis traced ancient EBV migrations and showed that recombinant EBV strains have undergone clonal expansion in East Asian populations (22). A new study of Southeast Asian patients with NPC showed that EBV of non-Chinese ancestry tended to carry fewer Southern Chinese NPC risk variants and clustered separately from both Chinese NPC and other EBV genomes (23). Together, these patterns suggest that EBV strain diversity is shaped by the host population history and geography, which, in turn, correlate with disease associations. The additional frequency comparison supports the phylogenetic observation that BBLF4-L322M is geographically structured. This variant was observed in East Asian data sets from Hong Kong, Guangxi, and Guangdong, but not in the African or European data sets. We therefore do not interpret BBLF4-L322M as a universal global prognostic marker. Instead, the current evidence is more consistent with a lineage-associated EBV variant that may have prognostic value within EBV-associated NPC in endemic southern Chinese populations. Its generalizability to other geographic settings remains to be established. Notably, the pooled public comparison did not show higher frequency of BBLF4-L322M in NPC than in healthy carriers. This suggests that BBLF4-L322M is unlikely to be an NPC-specific susceptibility mutation. However, prognostic association and disease specificity are distinct questions. A viral variant may be common in the background population yet still stratify clinical outcome within patients who develop EBV-associated NPC. We therefore interpret BBLF4-L322M more narrowly as a candidate prognostic marker in our Guangxi NPC cohort rather than as a disease-defining EBV alteration. This study had several limitations. First, our patients were recruited from a single endemic center, and the present findings require validation in independent, prospective, and multi-center cohorts. Second, because all enrolled cases had evidence of EBV infection, the prognostic association reported here should be interpreted as specific to EBV-associated NPC; EBV-negative NPC was not examined in this study. Third, we did not assess interactions with host genetics, including HLA variation, or tumor somatic alterations, and we lack direct functional data on the biological effects of L322M. Moreover, although we compared BBLF4-L322M across public EBV genomes from different regions and disease categories, these groups were not geographically matched. In particular, the lymphoma/BL data sets were predominantly African/European, whereas BBLF4-L322M was observed only in East Asian data sets. Therefore, the current comparison does not establish disease specificity and is strongly confounded by regional lineage background. The lack of healthy controls from Guangxi further limits interpretation. Accordingly, BBLF4-L322M should be regarded as a candidate region-linked prognostic marker rather than an NPC-specific EBV mutation. In conclusion, we identified BBLF4-L322M as a candidate region-linked EBV variant independently associated with poorer survival in EBV-associated NPC from Guangxi. These data extend the role of viral genetics beyond disease susceptibility and highlight actionable research directions. Integration of viral genotyping with clinical covariates may refine risk stratification, and mechanistic studies of BBLF4 are warranted to elucidate virus–host interactions in NPC. EBV sequence variations can affect the clinical trajectory of NPC, and our finding of BBLF4 L322M as a poor survival marker provides a new perspective on the role of the virus in cancer progression.

## MATERIALS AND METHODS

### Patient cohort and sample collection

This study included 114 patients with nasopharyngeal carcinoma (NPC). The inclusion criteria were as follows: (i) pathologically confirmed NPC, (ii) age ≥18 years and ≤80 years, (iii) no prior treatment before the diagnostic biopsy, (iv) evidence of EBV infection, and (v) availability of at least 5-year follow-up data after the initial treatment. Tumor biopsy specimens were obtained from each patient at diagnosis, and clinical data, including age, sex, and disease stage, were recorded. According to the available clinical records, none of the 114 patients had documented chronic immunodeficiency, autoimmune disease requiring systemic immunosuppressive therapy, organ transplantation, or another active malignancy at baseline. All the tissue samples were flash-frozen and stored at −80°C. Written informed consent was obtained from all the participants.

### Probe capture sequencing

DNA was extracted from tissue biopsies using an AllPrep DNA Mini Kit (Qiagen, Hilden, Germany) according to the manufacturer’s instructions. EBV-targeted enrichment was performed using the Agilent SureSelectXT Custom panel comprising 1,428 unique 120 nt biotinylated oligonucleotide bases tiled across the 171,321 bp EBV genome (NC_007605.1, GenBank; Table S4). Libraries were prepared according to the Illumina guidelines. DNA was ultrasonically sheared to 100–500 bp, end-repaired, A-tailed, adapter-ligated, and pre-capture amplified with a high-fidelity polymerase (10–12 cycles) to ensure yield while limiting bias. Hybridization was performed at 65°C overnight and bait–library hybrids were recovered with Dynabeads MyOne Streptavidin T1 magnetic beads, followed by stringent washes to remove nonspecific products. Post-capture PCR (8–12 cycles) was performed. Captured libraries were quantified using a Qubit 3.0 (Thermo Fisher Scientific, MA, USA) and assessed on an Agilent 2100 Bioanalyzer; only libraries with >5  ng/µL DNA and a modal insert size of 300–400 bp were advanced. Qualified libraries were equimolar pooled and sequenced on Illumina HiSeq/NovaSeq instruments using 2 × 150  bp paired-end chemistry to generate FASTQ files for downstream analyses.

### Variant calling and annotation

Raw reads were assessed using FastQC v0.11.5 (pre-filter mean *Q*_30_ = 93.90%). Adapters and low-quality bases were removed with fastp v0.20.1 (default settings) and Trimmomatic v0.39 to strip Illumina adapters. Clean reads were aligned to the EBV reference (NC_007605.1) using BWA-MEM v0.7.17. SAM files were converted to sorted, indexed BAMs with SAMtools v1.16.1. PCR duplicates were marked using Picard v2.27.1. Per-sample metrics showed a mean breadth ≥10× of 93.95% and a mean depth of 1,012× (range = 128–6,320×). Depth and ≥10× breadth distributions are summarized in Fig. 3E and F. Variant discovery followed GATK Best Practices (GATK v4.2.2.0): HaplotypeCaller (gVCF mode) per sample, and then CombineGVCFs/GenotypeGVCFs to produce a cohort VCF. Biallelic SNPs were subset and hard-filtered (SOR > 3.0, QUAL < 50.0, QD < 2.0, MQ < 40.0, FS > 60.0, MQRankSum < −12.5, and ReadPosRankSum < −8.0), yielding 6,035 candidates used for Base Quality Score Recalibration (BQSR). After recalibration, HaplotypeCaller was rerun. Sites within annotated repeats and ±5 bp were masked, positions with depth ≤15 were removed, and VCFs were merged. The final call set comprised 5,683 high-confidence biallelic SNPs across the EBV genome, with sample counts ranging from 868 to 1,629. Variants were annotated against the NC_007605.1 gene models to derive the genomic context and coding consequences.

### Phylogenetic analysis

We assembled a comparative EBV whole-genome panel of 731 sequences comprising 114 in-house strains and 617 public genomes. Public data were drawn from Hong Kong cohorts HK1 (PRJNA480052, https://www.ncbi.nlm.nih.gov/bioproject/PRJNA480052; 142 healthy saliva and 62 NPC biopsies) and HK2 (PRJNA577485, https://www.ncbi.nlm.nih.gov/bioproject/?term=PRJNA577485; 39 healthy saliva and 61 NPC biopsies), the GD data set (PRJNA522388, https://www.ncbi.nlm.nih.gov/bioproject/?term=PRJNA522388; 47 healthy saliva and 156 NPC biopsies) from Guangdong, African endemic (PRJEB38735, https://www.ncbi.nlm.nih.gov/bioproject/?term=PRJEB38735; 37 Burkitt lymphoma and 28 healthy), and European sequences (PRJNA505149, https://www.ncbi.nlm.nih.gov/bioproject/?term=PRJNA505149; 35 Burkitt lymphoma and 1 healthy) with additional European genomes from Palser et al. (PRJEB2768, https://www.ncbi.nlm.nih.gov/bioproject/?term=PRJEB2768; *n* = 9). Read processing, alignment to NC_007605.1, and cohort-level variant calling followed the variant calling and annotation pipeline described above, ensuring identical QC and filters across in-house and public data. After joint genotyping and hard filtering, 15,124 biallelic sites (SNPs + indels) were retained, and samples with <50% site retention (<7,562 sites) were excluded (*n* = 11), yielding a final set of 720 genomes (114 in-house  + 606 public) for tree reconstruction. Masking was applied to annotated repetitive regions (±5 bp) and sites with depth <15. Per-sample consensus, FASTA sequences were generated (bcftools v1.13) and aligned with MAFFT v7, and a maximum-likelihood phylogeny was inferred using IQ-TREE v2 under GTR + G with 1,000 bootstrap replicates rooted in the EBV reference (NC_007605.1). Tip metadata were annotated by geography (GX, GD, HK, Africa, and Europe) and phenotype (NPC, BL, healthy, and lymphoma) for downstream interpretation (Fig. 5).

### Statistical analysis

For the multivariate survival analysis, we constructed a Cox proportional hazards model, including the two EBV index variants identified (BZLF1 A205S and BBLF4 L322M), along with key clinical covariates. The clinical variables considered were age at diagnosis, sex, tumor stage, regional lymph node involvement, and distant metastasis status. To avoid small subgroup sizes, disease stage (per the AJCC 8th edition) was dichotomized into early-stage (Stages I–III) versus advanced-stage (Stage IV) NPC for modeling. Nodal involvement (cervical lymph node metastasis) and distant metastases (M1 disease) were included as separate binary covariates. The multivariate Cox model was used to estimate the independent effect of each EBV variant on overall survival while adjusting for these clinical factors. Hazard ratios (HRs) with 95% confidence intervals and Wald test *P* values were calculated for each variable in the model. All the statistical tests were two-sided, and a *P* value < 0.05 was considered statistically significant. Statistical analyses were performed using R software (version 4.2.0) and the survival package for Cox regression. Hierarchical clustering of variants was performed using Euclidean distance and average linkage criteria.

## SUPPLEMENTAL MATERIAL

Table S1 spectrum.02927-25-s0001.xlsx EBV strain genome sequencing quality metrics of EBV strains.

Table S2 spectrum.02927-25-s0002.xlsx Representative EBV genes with greater than or equal to 10 independent variants and their functional categories.

Table S3 spectrum.02927-25-s0003.xlsx Nonsynonymous EBV SNPs associated with overall survival in NPC patients.

Table S4 spectrum.02927-25-s0004.xlsx 1,428 biotinylated oligonucleotide probes covering the 171,321 bp EBV genome.

ASM does not own the copyrights to Supplemental Material that may be linked to, or accessed through, an article. The authors have granted ASM a non-exclusive, world-wide license to publish the Supplemental Material files. Please contact the corresponding author directly for reuse.
